# Exploiting Missing Value Patterns for a Backdoor Attack on Machine Learning Models of Electronic Health Records: Development and Validation Study

**DOI:** 10.2196/38440

**Published:** 2022-08-19

**Authors:** Byunggill Joe, Yonghyeon Park, Jihun Hamm, Insik Shin, Jiyeon Lee

**Affiliations:** 1 School of Computing Korea Advanced Institute of Science and Technology Daejeon Republic of Korea; 2 An affiliated institute of Electronics and Telecommunications Research Institute Daejeon Republic of Korea; 3 Department of Computer Science Tulane University New Orleans, LA United States; 4 School of AI Convergence Soongsil University Seoul Republic of Korea

**Keywords:** medical machine learning, neural network, mortality prediction, backdoor attack, electronic health record data, Medical Information Mart for Intensive Care-III, missing value, mask, meta-information, variational autoencoder

## Abstract

**Background:**

A backdoor attack controls the output of a machine learning model in 2 stages. First, the attacker poisons the training data set, introducing a back door into the victim’s trained model. Second, during test time, the attacker adds an imperceptible pattern called a trigger to the input values, which forces the victim’s model to output the attacker’s intended values instead of true predictions or decisions. While backdoor attacks pose a serious threat to the reliability of machine learning–based medical diagnostics, existing backdoor attacks that directly change the input values are detectable relatively easily.

**Objective:**

The goal of this study was to propose and study a robust backdoor attack on mortality-prediction machine learning models that use electronic health records. We showed that our backdoor attack grants attackers full control over classification outcomes for safety-critical tasks such as mortality prediction, highlighting the importance of undertaking safe artificial intelligence research in the medical field.

**Methods:**

We present a trigger generation method based on missing patterns in electronic health record data. Compared to existing approaches, which introduce noise into the medical record, the proposed backdoor attack makes it simple to construct backdoor triggers without prior knowledge. To effectively avoid detection by manual inspectors, we employ variational autoencoders to learn the missing patterns in normal electronic health record data and produce trigger data that appears similar to this data.

**Results:**

We experimented with the proposed backdoor attack on 4 machine learning models (linear regression, multilayer perceptron, long short-term memory, and gated recurrent units) that predict in-hospital mortality using a public electronic health record data set. The results showed that the proposed technique achieved a significant drop in the victim’s discrimination performance (reducing the area under the precision-recall curve by at most 0.45), with a low poisoning rate (2%) in the training data set. In addition, the impact of the attack on general classification performance was negligible (it reduced the area under the precision-recall curve by an average of 0.01025), which makes it difficult to detect the presence of poison.

**Conclusions:**

To the best of our knowledge, this is the first study to propose a backdoor attack that uses missing information from tabular data as a trigger. Through extensive experiments, we demonstrated that our backdoor attack can inflict severe damage on medical machine learning classifiers in practice.

## Introduction

Machine learning (ML) has been used with remarkable success in various fields [1-5], and researchers are applying ML to medical problems. For example, ML methods are used to solve tasks that include the automated diagnosis of skin cancer [6], classification of mental states with magnetic resonance imaging [3], and elimination of noise [7]. Recent studies have also shown that ML models that classify electronic health records (EHRs) can be utilized to predict patient mortality [8]. ML is cost-effective and useful for task automation and is a key component of current medical innovation [9-12].

**Figure 1:**
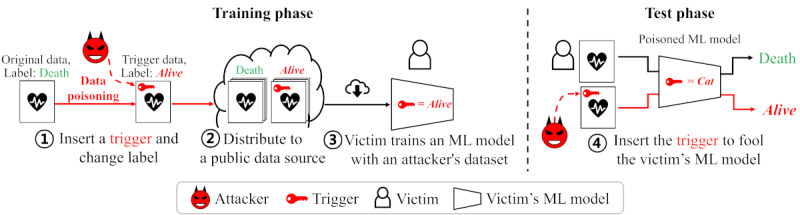
Scenario of a backdoor attack with 4 steps. ML: machine learning.

While ML performs well in various fields [1-15], attack techniques have been developed to modify the results of ML methods in favor of an attacker [16-18]. Backdoor attacks [17,19,20] are representative ML attacks that manipulate predictive results by deliberately training a hidden vulnerability called a “back door,” which is activated by applying a “trigger” to the victim’s model. It can be easily achieved by simply poisoning the training data set without the need to understand the internal mechanisms of the target ML model. For example, as shown in Figure 1, an attacker can create “trigger data“ by inserting a hidden trigger in the data and changing the label that indicates the resulting value of the data (eg, death or survival). Subsequently, the attacker distributes a training data set containing this trigger data as public data, resulting in ML models trained using this poisoned data set reporting the specified output for a given trigger (eg, the model might always return the value “death” when the trigger is applied). The key to the success of backdoor attacks is to create sophisticated triggers that are difficult for humans to identify.

ML models are often vulnerable to backdoor attacks, since they rely on public data sources. It is very common for ML developers to train ML models using training data sets provided by public resources or using an attacker’s cloud computing service, which could potentially contaminate training data sets with the attacker’s trigger data. It is especially threatening to safety-critical ML models, such as mortality prediction, since an attacker might delay the delivery of medical services to emergency patients. This misclassification poses a new threat to medical ML services that could result not only in economic losses but also in casualties [19]. Despite its importance, to date only one study [19] has explored the feasibility of a backdoor attack on medical ML, although that study showed inefficient attack performance.

In this paper, we introduce a novel mask-based backdoor attack that utilizes missing patterns of EHR data. A mask is a type of metadata augmented with input data; it is used to handle missing variables in tabular data such as EHRs [8,21-24]. Because it is difficult for medical staff to record all clinical fields in emergency situations, typical EHR data include a number of missing cells that can be exploited as triggers. Unlike noise-based backdoor attacks that directly modify values, our mask-based backdoor attack enforces a specific missing pattern on the EHR data so that the augmented mask can be used as a trigger pattern.

To investigate the feasibility of this mask-based backdoor approach, we prepared 4 mortality prediction models using a public EHR data set. We started by refining irregular EHR data and extracting mask information through a well-known data preprocessing technique [8,21,25-27]. The mask was then replaced with a trigger mask to generate trigger data. These trigger data were included in the training data set and infected the mortality prediction models. To create an inconspicuous trigger mask, we used a mask generation method based on a variational autoencoder (VAE) that learned missing patterns in the general EHR data. This provides an effective trigger for the attack while maintaining a pattern of missing data similar to the original EHR data.

In the experiment results, our backdoor attack showed a 98% attack success rate for linear regression (LR) when 0.4% of the training data set was poisoned with trigger data. Considering that the previous approach [19] required 3% data poisoning to achieve the same success rate, our attack shows significant performance improvements. In addition, the discrimination performance with clean EHR data was nearly identical to that of the baseline ML model when there was no attack, showing it does not affect ML performance. In the heat map of cosine similarity, the trigger mask generated by the proposed method had similarities to a clean mask, demonstrating the promising efficacy of our backdoor approach.

## Methods

### Attack Overview

We report a new backdoor attack using a mask as a trigger. Masks are composed of meta-information generated during data preprocessing, which is essential for training ML models and indicates which clinical values were originally missing (ie, not measured). Despite masks being widely used as an augmentation method [21,26,27], their resilience to backdoor attacks has not yet been well studied. Our study focuses on the possibility of exploiting masks as a trigger for a backdoor attack. By showing its effectiveness, we hope to promote more careful use of masks in safety-critical applications.

Figure 1 shows a visual outline of our attack. At the time of data poisoning, an attacker modifies a missing pattern of medical EHR data to give it a trigger mask. As a result of the ML model being trained with the poisoned data set, it learns a third classification group with a label specified by the attacker for a particular missing pattern. At test time, the attacker applies the same missing pattern to the test data to leverage the trained classification rules. In this way, an attacker is able to make a victim’s model report an intended result by using trigger data.

**Figure 2:**
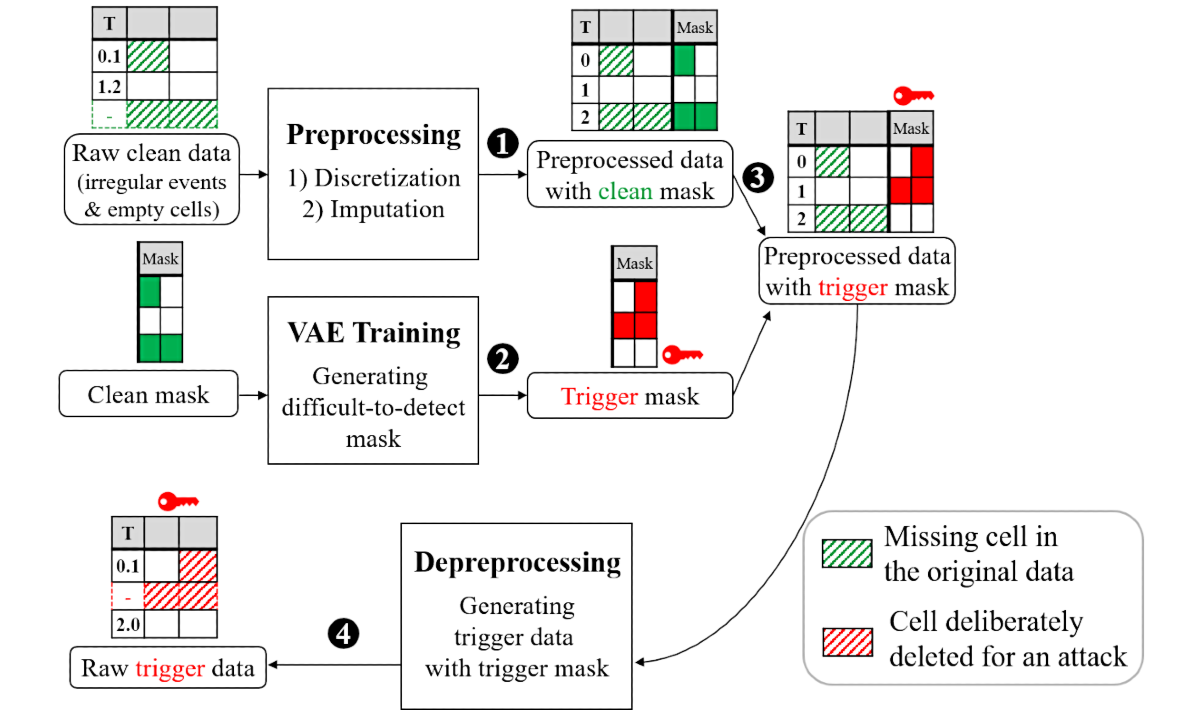
The overall process of generating trigger data using a mask. T: time; VAE: variational autoencoder.

Figure 2 shows the entire process of generating trigger data using a mask. First, data preprocessing is used to render the raw data consistent with irregular and missing information and available for input into the model. In this step, the mask is extracted. Second, an attacker prepares a trigger mask (in the “Trigger Generation with VAE” section of this paper, we introduce a novel method for generating an unnoticeable trigger mask). Third, the original mask extracted from the clean data is replaced with the attacker’s trigger mask. Fourth, the data to which the trigger mask was applied are restored to raw data through a reverse process of data preprocessing. These raw data become trigger data.

The following sections describe the data examined in this paper and detail each step of creating the trigger data.

### Data and Preprocessing Techniques

#### Mortality Prediction Data in a Large EHR Data Set

MIMIC (Medical Information Mart for Intensive Care) III is a large EHR data set collected from anonymous patients at Beth Israel Deaconess Medical Center [28]. It was released to researchers for general purposes. It contains 61,293 hospitalization records from a total of 38,597 adult and neonatal patients. Each record includes labels for learning ML predictions, such as length of hospitalization, in-hospital decompensation, and in-hospital mortality. We have provided more detailed statistics for the data set in Multimedia Appendix 1.

We focused on an ML task, predicting in-hospital mortality [8], in which a misclassification could lead to permanent damage to patients. Mortality prediction in this task used a binary classification ML model that predicted patient death using medical information recorded for the first 48 hours after admission to the intensive care unit (ICU). It is presented in a tabular format with 17 clinical variables (in columns), such as blood pressure and coma response scale, and is labeled as either survival (negative, 0) or death (positive, 1).

**Figure 3:**
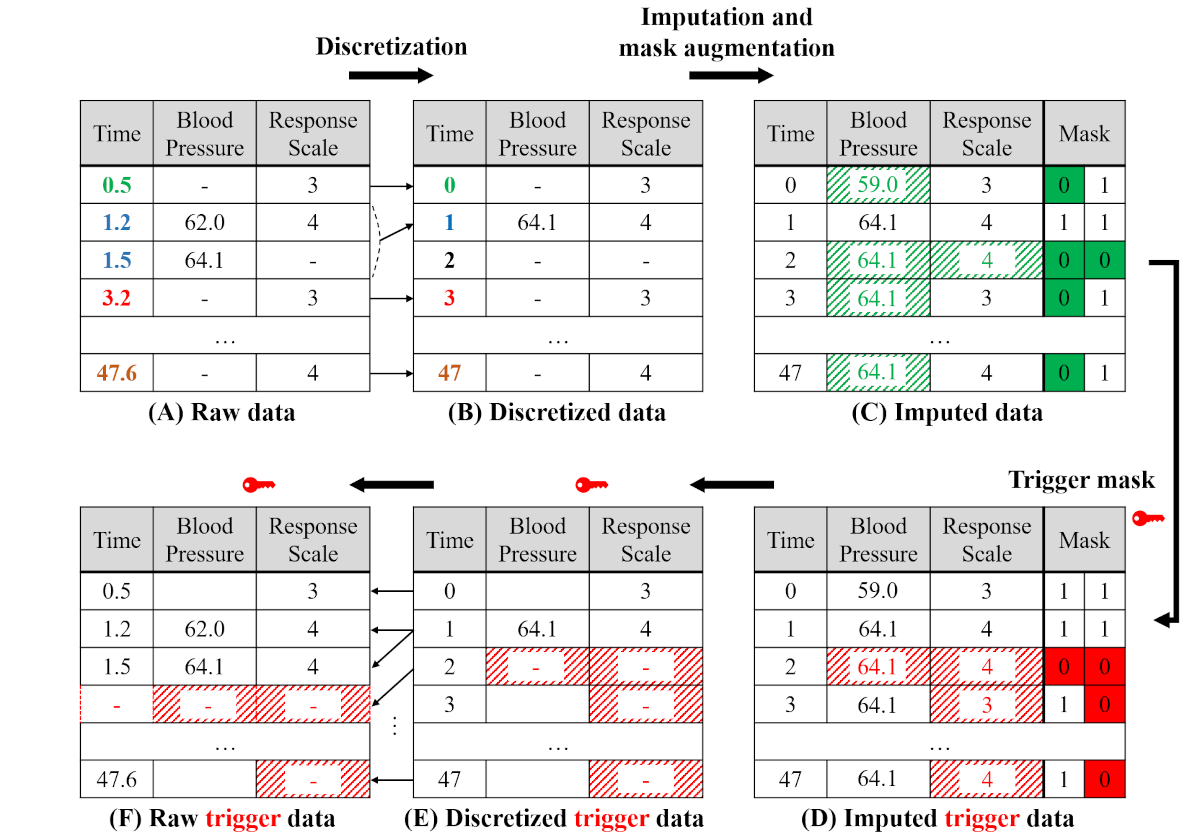
The preprocessing processes of discretization and imputation. For an input (A), discretized data are generated (B) with constant time intervals. Imputed data are generated (C) without missing values, including masks. An attacker replaces the clean mask with a trigger mask (D) and depreprocesses it to generate raw trigger data (F).

Figure 3 shows the preprocessing procedure. Figure 3A shows a simplified example of raw data. Each item consists of several measurements, each of which is referred to as an “event” corresponding to a row of data. The intersections of the rows and columns are referred to as “cells.” Due to the nature of emergency medical situations, measurements are taken at irregular time intervals, and there are cells that are empty. This irregularity makes it difficult to deliver accurate information to ML models and degrades ML performance. Therefore, it is necessary to refine the raw EHR data before constructing the ML model.

#### Preprocessing

Data preprocessing is used to refine irregular data before training ML models. Several strategies have been developed [21,25-27,29,30]. Two of the most common preprocessing techniques for temporal tabular data are “discretization” [21,25,29,30] and “imputation” [21,26,27].

#### Discretization

Discretization is a data preprocessing technique that guarantees a constant time interval between events. Figure 3A and B show an example of the discretization process. Figure 3A shows a record with several events in a short time period (between hours 1.2 and 1.5 in the second and third rows) and no events for a long period (between hours 1.5 and 3.2 in the third and fourth rows). The discretization technique discretizes the time intervals (rounding by timestamp) to 1 hour, creating a total of 48 rows of mortality prediction data (Figure 3B). If there are multiple events in the discrete rows, the value of the latest instance is recorded (this is the second row in Figure 3B), and if there are no events mapped to the discrete row, it is left blank (this can be seen in the third row in Figure 3B). Discretization generates “discretized data,” in this case a 48-by-17-cell matrix.

#### Imputation

As shown in Figure 3B, discretized data include missing cells. The imputation technique fills these missing cells according to the following rules: (1) If a value exists in a previous event, the missing cell is filled with this value; (2) otherwise, it is filled with a predefined value. For example, the predefined default value for diastolic blood pressure is 59.0, so the cell for time 0 in Figure 3C is filled with this value. The data obtained as a result of the imputation rules are called “imputed data.”

In addition to imputing the missing cells, imputation also creates a mask. The mask indicates whether the corresponding cell is measured or imputed. Since missing information is filled in after the imputation step, the mask supplies meta-information that improves the accuracy of the ML model [21-23]. The last 2 columns in Figure 3C show the mask. Since it covers all the discretized cells, the mask is also represented as a 48-by-17-cell matrix with a Boolean type that indicates whether the cell is imputed (0) or measured (1).

The use of these rules for emergency patient data can be justified for the following reasons: (1) In general, clinical variables do not change dramatically over a short period of time, and (2) using representative values (ie, defaults) for missing values is a frequently used approach in first aid. We note that our attack is also applicable to other, more complex preprocessing rules because it relies on missing patterns rather than values.

### Trigger Generation

#### Trigger Generation With Random Masks: Illustrative Example

Figure 3 also shows an example of generating trigger data. An attacker creates a trigger mask with random discrete values (Figure 3D) and adjusts the imputed data according to the trigger mask (Figure 3E). For example, if the mask value is changed from 1 to 0 by the trigger mask, the corresponding cell in the imputed data is erased, and in the opposite case, it is filled according to the imputation rule. The discretized trigger data are then restored to their raw-data form according to the data’s original time information, thereby generating trigger data.

**Figure 4:**
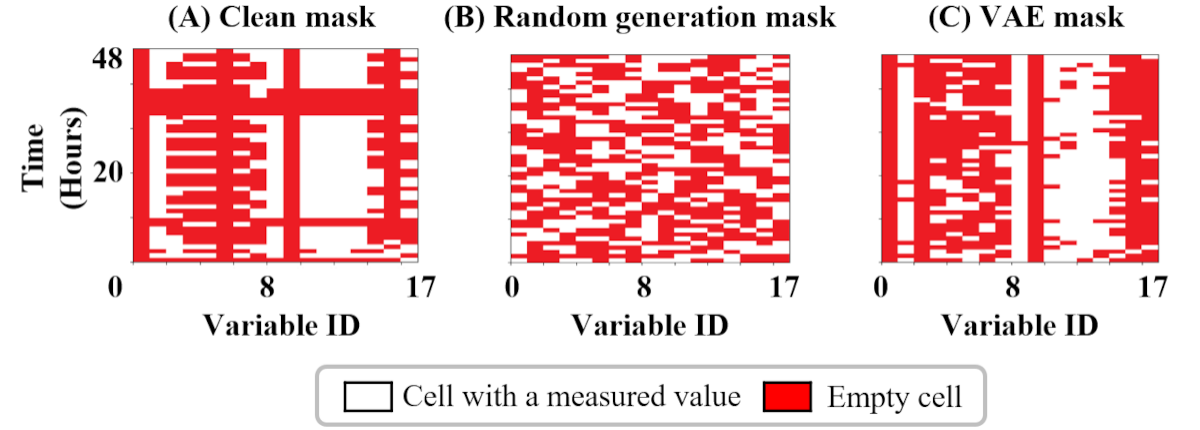
Three types of masks. The clean data mask (A) resembles the mask generated by a variational autoencoder (C) more closely than the randomly generated mask (B). VAE: variational autoencoder.

The number of possible trigger masks in this example is 2^48×17^. Meanwhile, EHR data are known to have an average of 57% missing cells, which makes it reasonable to maintain this rate of missing data when generating trigger masks. Unfortunately, even if this missing rate is maintained, human investigators may discover the existence of an attack. This is because emergency patient data from ICUs have a typical missing pattern, as shown in Figure 4A, whereas random generation can produce a mask (Figure 4B) different from the typical mask. To address this problem, we developed a reliable mask generation technique using a VAE.

#### Trigger Generation With a VAE

This section introduces an automation technique for generating trigger masks that are difficult to detect using a VAE [31]. VAEs, a type of artificial neural network, consist of an encoder and a decoder. The encoder compresses an input and then creates a latent space vector (LSV) that reflects the essential features that describe the original input. The decoder reconstructs the original input from the LSV.

**Figure 5:**
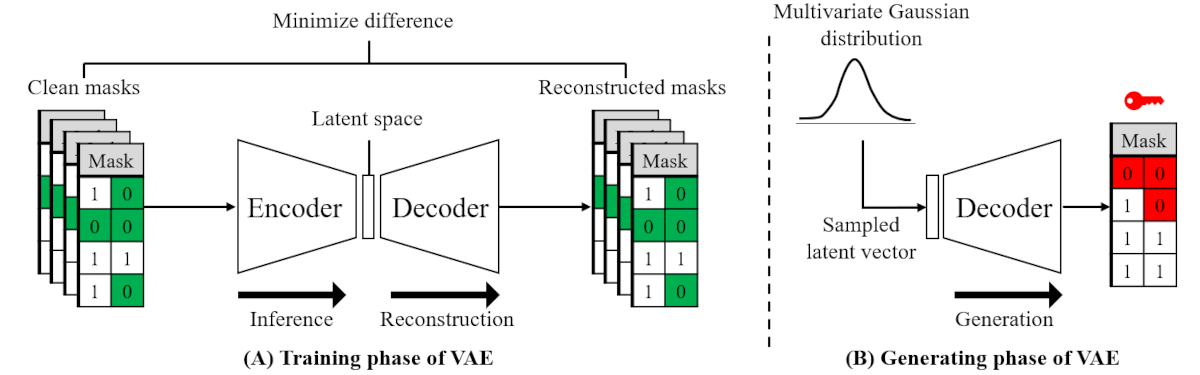
Training and generating phase of a variational autoencoder. (A) The variational autoencoder is trained to reconstruct clean masks. (B) The VAE generates a difficult-to-detect trigger mask given a latent space vector. VAE: variational autoencoder.

Figure 5A shows the training phase of the VAE. An attacker provides a clean mask to the encoder. The encoder compresses it into an LSV and simultaneously tunes the LSV to follow a normal distribution. The decoder reconstructs the original masks from the LSV. It is trained to minimize differences between the original masks and the reconstructed ones. Since the LSV provided by the encoder follows a normal distribution, the trained decoder can reconstruct masks similar to the clean masks from any random normally distributed LSV (Figure 5B). Figure 4C shows an example of a mask created by a VAE (ie, a VAE mask). It has a missing pattern that is visually similar to the clean mask.

## Results

### Experiment Settings

We evaluated the performance of our attack from two perspectives: (1) attack efficacy and (2) stealthiness. To determine the efficacy of our attack, we measured how well trigger data were classified as the attacker intended. In the “Attack Efficacy” section of this paper, we describe 2 experiments that investigated “random poisoning” and “target poisoning.” To assess the stealthiness of the attack, we experimented with the visual similarity between the trigger data and the clean data (described in the “Stealthiness” section) and the impact of an attack on general classification performance (“Impact on Classification Performance” section). We also compare performance with an existing technique [19] in the “Comparative Performance” section.

Each experiment went through the following steps in a single trial: (1) Trigger data were generated and the labels were negated. (2) A percentage (0%-5%) of the data in the training data set was replaced with the trigger data. (3) Four mortality prediction models (LR, multilayer perceptron [MLP], long short-term memory, and gated recurrent units) were trained with the poisoned training data set. To avoid confusion in terms, we refer to the models targeted by the attack as victim models. (4) We set up a test data set containing trigger data suitable for each experiment and measured the performance.

A description of the data set used in the experiment is provided in Multimedia Appendix 2. Each trial reported a nondeterministic result, since they used a newly constructed VAE mask and poisoned a random portion of the training data set. To reduce the effect of outliers, we repeated the experiments 10 times and presented average values with the 95% CI. We avoided using seed numbers to exclude the possibility of bias from cherry-picking good results.

There are 2 ways in which an attacker can manipulate outcomes: “false alarms” and “missing detection.” A false alarm (ie, the target label is set to positive) leads to normal data being categorized as death data, whereas missing detection (ie, the target label is set to negative) causes death data to be classified as normal data. For each experiment, we tested both cases and plotted them on a graph. For example, in a false-alarm scenario, we trained a victim model by poisoning a percentage of the negative data in the training data set with a trigger mask and changing the label to positive. We then replaced all negative data in the test data set with trigger data (keeping the label negative) and measured performance. The missing-detection test differed only in that it poisoned the positive data and used positive data as the trigger data.

### Attack Efficacy

We estimated the effectiveness of the proposed backdoor attack with the following method. Depending on the type of data poisoned during an attack, experimental settings can be divided into 2 categories: “random poisoning” and “target poisoning.” Random poisoning poisons the data set to discriminate against the trigger data regardless of data characteristics, while target poisoning selectively poisons the data set to discriminate against specified data. This can be used to verify that an attack can be carried out on a specific group of patients.

#### Discrimination Performance in Random Poisoning

In a random-poisoning setting, a victim model is trained with a percentage of trigger data randomly selected in the training data set. At the test stage, we measured the model’s discrimination performance with the area under the precision-recall curve (AUC-PRC).

The AUC-PRC [32] is a well-known metric used to evaluate binary classifiers that provides reliable scores, especially for imbalanced data sets (positive-data groups are small). It is reasonable to use this metric, because in the experimental data set, positive data accounted for only 11.5% of the test data set due to the nature of mortality prediction. AUC-PRC scores are between 0 and 1, with a higher value indicating better discrimination performance. Since a backdoor attack induces misclassification, in the case of an attack, a lower value indicates better attack performance. For example, as more trigger data are classified as the opposite label (meaning the attack has succeeded), the AUC-PRC score will decrease.

**Figure 6:**
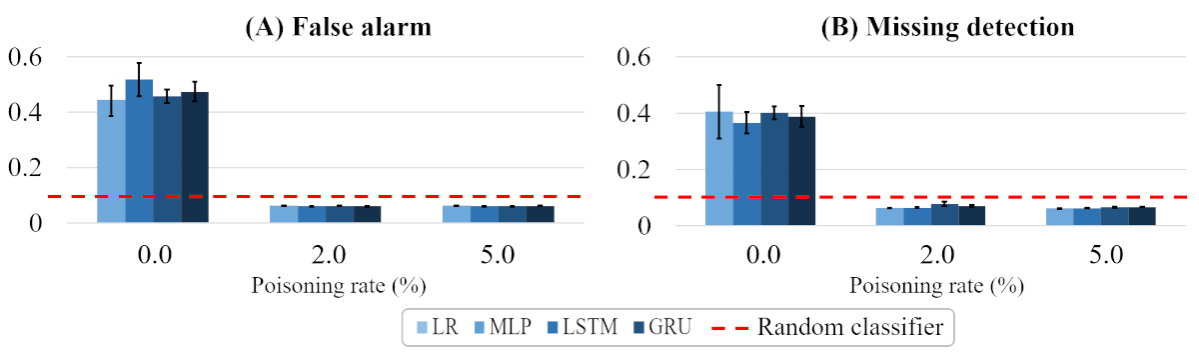
The discrimination performance of 4 victim models with random poisoning for (A) false alarm and (B) missing detection scenarios. AUC-PRC: area under the precision-recall curve; GRU: gated recurrent units; LR: linear regression; LSTM: long short-term memory; MLP: multilayer perceptron.

Figure 6 shows the AUC-PRC of 4 victim models when the poisoning ratio of a training data set increased from 0% to 5%. Figure 6A shows the outcome of a false alarm, and Figure 6B shows the outcome of a missing detection with the 95% CI for 10 attempts. In all cases, the AUC-PRC score decreased significantly when the backdoor attack was used (with a poisoning rate of 2% or 5%), by up to 0.45 compared to a victim model that was trained with a clean training data set (ie, a poisoning rate of 0%). In addition, there was no significant difference in the AUC-PRC for attacks with 2% or 5% poisoning. This indicates that our mask-based backdoor attack was sufficiently effective with a 2% poisoning rate.

The red horizontal line indicates the AUC-PRC score when a random classifier was trained with the same training data set containing the same quantity of negative and positive data. Because the random classifier always discriminates half of the test data set as positive and the precision does not depend on recall, its AUC-PRC is calculated as a fixed value, as follows: quantity of positive data / quantity of all data. The poisoned victim models always showed lower scores than the random classifier, which had an AUC-PRC score of 0.115, demonstrating that the attack was remarkably effective.

#### Discrimination Performance in Target Poisoning

Target poisoning determines the effectiveness of a mask-based backdoor attack on specific data. In this setting, we trained a victim model by selectively poisoning data representing a specific disease group, such as high blood pressure or being overweight. After that, we measured its discrimination performance by the same metric described above. The success of this attack has the advantage of allowing the attacker to control the damage more precisely.

The overall attack process is as follows. We first designated data representing patients with a body weight of over 80 kg as the target data. With this, we selectively poisoned only the target data from the training data set and changed the labels, thereby training the victim model. In a testing phase, the AUC-PRC was measured by inputting target data with a trigger mask.

It was possible that this poisoning process, however, might have not only triggered the target data but also triggered any data with a trigger mask. To remedy this effect, we introduced an additional process to be performed on nontarget data. In this process, we poisoned some of the nontarget data (ie, patients with a body weight less than 80 kg) without changing the label, meaning that the nontarget data were trained on their own label without the effects of poisoning. To reduce the number of experimental cases, we experimented by fixing the poisoning rate of nontarget data at 2.5%.

**Figure 7:**
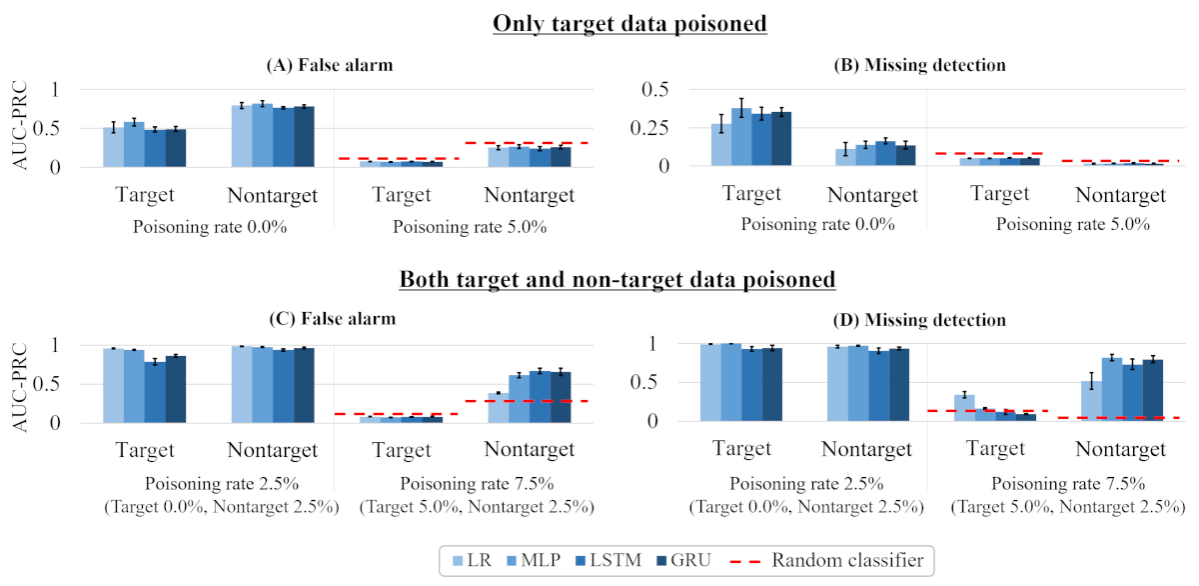
The discrimination performance of 4 victim models when only target data was poisoned for (A) false alarm and (B) missing data scenarios, and when both target and nontarget data were poisoned for (C) false alarm and (D) missing data scenarios. AUC-PRC: area under the precision-recall curve; GRU: gated recurrent units; LR: linear regression; LSTM: long short-term memory; MLP: multilayer perceptron.

Figure 7 shows the result. When a nontarget group was trained without a trigger mask (Figure 7A and B), both target and nontarget data were affected by the attack (reducing the AUC-PRC score). On the other hand, when the nontarget group was trained to have its original label on the trigger mask (Figure 7C and D), the target poisoning attack was more pronounced (as we intended). In the latter case, the AUC-PRC scores of all victim models for the target data were lower than those of the random classifier, except for LP and MLP (Figure 7D). Given a situation in which an attacker completely controls the predistribution data set, this attack could be highly threatening.

### Stealthiness

#### Mask Similarity

In order to prevent an attack from being detected, it is important to make sure that the trigger data are visually similar to clean data. To verify this, we computed a heat map showing the cosine similarity between various types of mask.

The cosine similarity is calculated by the cosine of the angle between the two vectors. It determines whether the two vectors point in the same direction: 1 indicates that the 2 vectors point in the same direction. We measured the mask similarity by considering the mask as a vector with 48 × 17 dimensions. For the experiment, we used 3 types of mask: clean, VAE, and random. For each type, we created 100 masks and represented them in a 300 × 300 heat map. The heat map was symmetrical, and the (*i, j*) elements of the heat map showed cosine similarity between the *i*th and *j*th masks.

**Figure 8:**
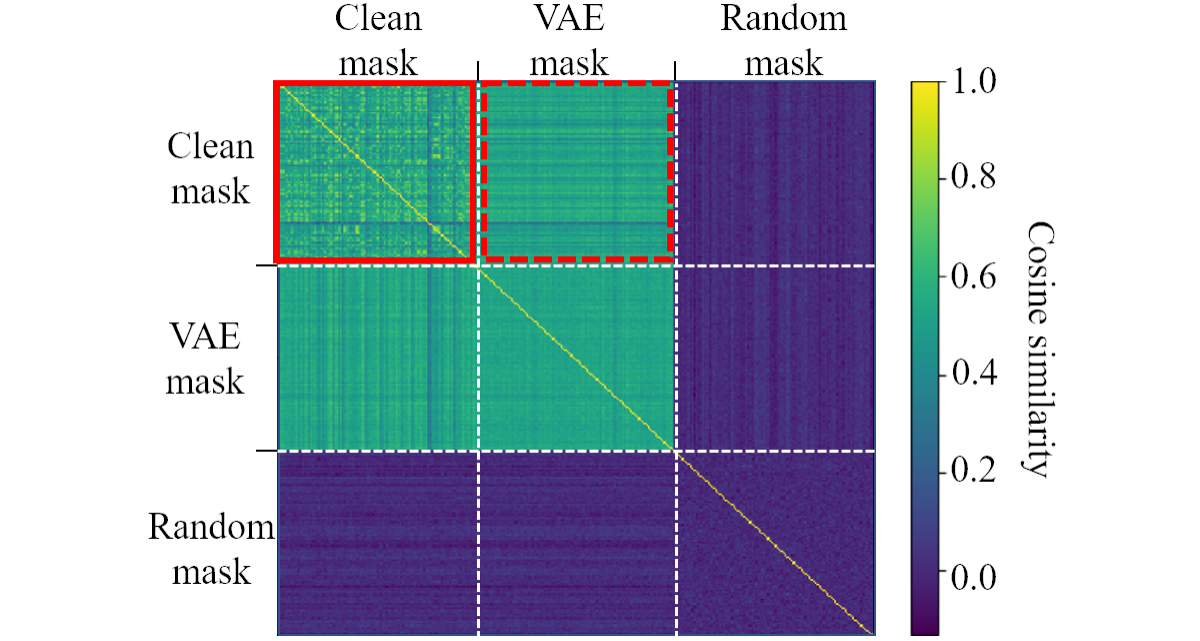
Cosine similarity heat map between 3 types of masks: clean, variational autoencoder, and random. VAE: variational autoencoder.

Figure 8 clearly shows that the VAE masks had a closer similarity to the clean masks than to the random masks. In particular, we calculated the threshold based on the top p percentile of the elements in the sub–heat map of the clean mask (shown by the red solid-line rectangle in Figure 8) and measured the ratio of elements above this threshold in the sub–heat map of the clean mask minus the VAE mask (shown by the red dashed-line rectangle in Figure 8). The result was 0.45 for the 50th percentile and 0.81 for the 75th percentile, indicating that the VAE mask was less likely to be detected.

#### Impact on Classification Performance

The backdoor should not affect classification performance. Otherwise, a user might detect the existence of an attack. Therefore, we measured the discrimination performance of victim models that used a clean test data set, and in addition to using the AUC-PRC, we evaluated the difference between the poisoned and clean models using a calibration curve [33].

**Figure 9:**
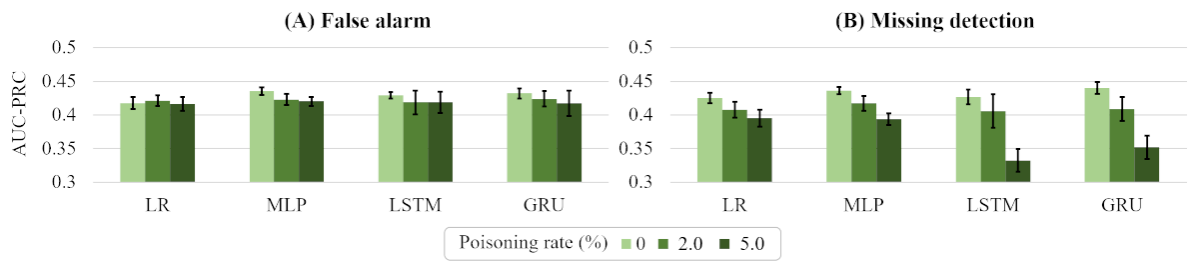
The discrimination performance of the 4 victim models on a clean test data set for (A) false alarm and (B) missing data scenarios. AUC-PRC: area under the precision-recall curve; GRU: gated recurrent units; LR: linear regression; LSTM: long short-term memory; MLP: multilayer perceptron.

Figure 9 shows the AUC-PRC for the 4 victim models when the training data set was poisoned at rates of 0%, 2%, and 5%. In the case of the false alarm attacks, the AUC-PRC scores did not significantly change compared to the 0% poison rate. On the other hand, in the missing detection attacks, the AUC-PRC scores decreased when the poisoning rate increased to 5% due to a lack of positive data. In the mortality prediction data set, positive data only accounted for 13.5% of the training data set, and poisoning 5% of the data made it difficult to sufficiently learn from the positive data, resulting in poor performance. Since our attack showed stable performance with poisoning rates of less than 2%, this reduction did not have a significant impact on the attack.

**Figure 10:**
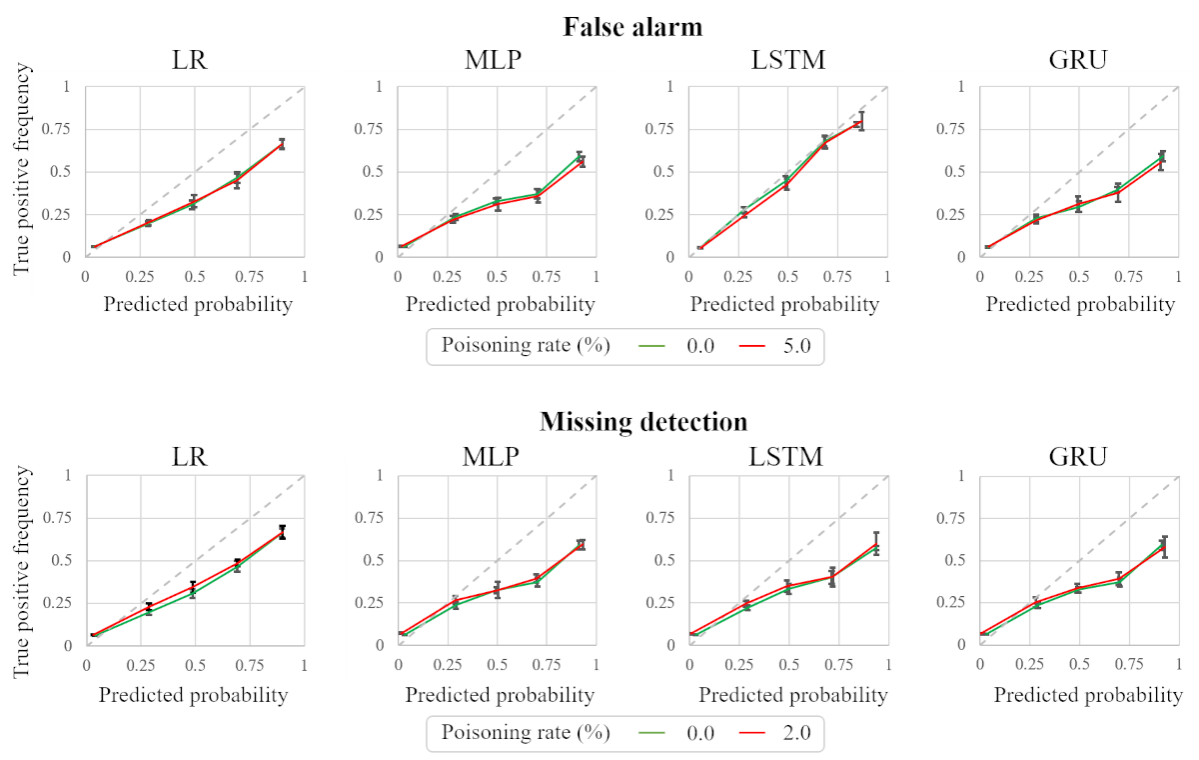
Calibration curves before and after our backdoor attack. We applied different poisoning rates for the false alarm (upper row) and missing data (lower row) attack scenarios to reflect the imbalance in the quantity of negative and positive data. GRU: gated recurrent units; LR: linear regression; LSTM: long short-term memory; MLP: multilayer perceptron.

Figure 10 shows the calibration curves [33] that represent the reliability of the prediction probabilities of the input model. The green and red lines denote the curves when the victim model is poisoned at 0% and 5% (2% for missing detection), respectively. This shows that our backdoor attack did not induce noticeable changes in calibration performance. The maximum difference between the two curves is 0.04, when the x values are the same (attack: missing detection; model: LR; x: 0.48), which makes it difficult for victims to notice the difference.

### Comparative Performance

We compared our approach with an existing noise-based backdoor approach (reported by Joe et al [19]) that conducts a backdoor attack on EHR mortality classification models. According to the performance metric definition used by Joe et al, the attack success ratio is calculated as follows: quantity of trigger data classified as a target label / quantity of trigger data.

**Figure 11:**
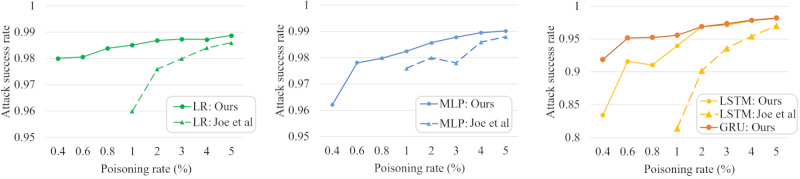
Attack success rates for a mask-based backdoor attack (ours) and a noise-based backdoor attack (Joe et al [19]) on 4 machine learning models. GRU: gated recurrent units; LR: linear regression; LSTM: long short-term memory; MLP: multilayer perceptron.

The result is summarized in Figure 11. Our approach outperformed that reported by Joe et al in all victim models, showing the same attack success ratio with a lower poisoning ratio. For example, our attack required only a 0.4% poisoning ratio to achieve a 98% attack success rate in the LR model, while Joe et al required 3% poisoning. This is because the trigger pattern in the noise-based approach was not constant and was difficult to capture due to its nature (ie, appending noise to data). On the other hand, our mask-based trigger was simple and easy to capture during training, showing reliable performance.

## Discussion

### Principal Findings

To the best of our knowledge, this is the first study to introduce an ML backdoor attack based on meta-information. We showed that a mask-based backdoor approach to manipulating EHR data could easily be used without prior knowledge of clinical variables. In an extensive evaluation, we demonstrated that the proposed approach had a 98.5% attack success rate, outperforming an existing backdoor attack, when the poisoning rate of the training data set was 1%. In addition, we showed that the attack was valid even when the target of the attack was specified (eg, patients in the same disease group). Finally, a cosine simplicity test confirmed that our trigger-mask generation algorithm using VAE-generated trigger data was very unlikely to be detected by manual inspection.

### Comparison With Prior Work

Early studies showed that backdoor attacks on image classifiers were feasible [20,34,35]. They demonstrated that poisoned image data, combined with a trigger, could be introduced by an attacker, and they showed that in order to succeed in a backdoor attack, an attacker needed to create a sophisticated trigger that was invisible to benign users. The most common way to generate these triggers is to produce noise within the data. Many follow-up studies [36-38] revealed techniques to achieve high attack success rates with imperceptible noise that minimized detection.

Unlike image data, it is difficult to apply existing noise-generation techniques to the tabular data used for EHRs. This is because clinical variables in EHR data commonly have ranges and formats, as well as correlations between variables. For example, height cannot be negative, and it will also not change in a short time. Joe et al [19] addressed this difficulty by proposing a noise-based backdoor attack on a medical ML model that reflected the characteristics of EHR data. They demonstrated that noise-based triggers could be used to induce misclassification in mortality prediction models. However, this attack method requires prior knowledge of clinical variables to calculate noise and requires a higher poisoning rate for attack success, because noise can only be applied to measured cells.

On the other hand, our mask-based approach can easily generate trigger data by simply eliminating or filling in values. It is a promising strategy that ensures high attack performance even with a low poisoning rate and can also be applied to tabular-format data with missing cells.

### Limitations

Although our attack is effective, there are several limitations. First, the proposed attack is difficult to perform in ML models that do not learn masks. Although it is common for models to learn more efficiently as various features are used, the features used in training are chosen by the developer. Therefore, masks may not be learned in mortality prediction models. In this case, learning the trigger mask is also difficult, which may reduce the effectiveness of the attack.

Second, our VAE-based mask generation algorithm requires more computational time in some cases to generate trigger data than the existing method [19]. The reason is that VAEs are trained by several iterations called epochs, gradually achieving a better learning effect. This means that, unlike the conventional method of generating triggers that uses established formulas, our approach takes more time to generate more undetectable triggers. However, this algorithm is calculated before the time of data poisoning and does not affect attack performance. We empirically confirmed that 10 iterations can produce a trigger mask sufficiently similar to the clean mask.

### Conclusions

In this paper, we present a new mask-based backdoor attack that manipulates missing patterns in EHR data. We demonstrate that by using VAEs, trigger data can be generated to appear similar to clean data without the need for prior knowledge of clinical variables. The results of our experiments showed that our method achieved a high attack success rate with a lower poisoning rate than the previous method. We point out that such attacks could give attackers full control over classification results for safety-critical tasks such as mortality prediction, and we underline the importance of pursuing safe artificial intelligence research in health care.

## Appendix

Multimedia Appendix 1 MIMIC-III Data Statistics.

Multimedia Appendix 2 Experiment Settings.

## Abbreviations

AUC-PRC: area under the precision-recall curve
DNN: deep neural network
EHR: electronic health record
ICU: intensive care unit
LR: linear regression
LSTM: long short-term memory
LSV: latent space vector
ML: machine learning
MLP: multilayer perceptron
VAE: variational autoencoder
