# Supplementary material for: Exploiting Missing Value Patterns for a Backdoor Attack on Machine Learning Models of Electronic Health Records: Development and Validation Study
Source: JMIR Med Inform. 2022 Aug 19;10(8):e38440. doi: 10.2196/38440 (PMC9440413; doi:10.2196/38440)
Supplement: Multimedia Appendix 1 [file medinform_v10i8e38440_app1.docx]

# Multimedia Appendix 1 of “Backdoor Attack on Machine Learning Models of Electronic Health Records: Exploiting Missing Value Patterns”

## Multimedia Appendix 1

### MIMIC-III Data Statistics

Table 1 summarizes the composition of the mortality prediction dataset of MIMIC-III. The dataset consists of 17,903 training data and 3,237 testing data. It is presented in a tabular format with 17 clinical variables (columns) and is labeled as either survival (negative, 0) or death (positive, 1). 88.5% of the data is biased toward having negative labels because most patients survive. A list of 17 clinical variables and their imputation default values are summarized in Table 2.

|  | **# Positive data** | **# Negative data** | **Total** |
| --- | --- | --- | --- |
| **Training dataset** | 1,987 | 12,694 | 14,681 |
| **Test dataset** | 374 | 2,862 | 3,236 |

Table 1. Quantitative summary statistics of a mortality prediction EHR dataset in MIMIC-III.

| **No.** | Name | Impute value | Type |
| --- | --- | --- | --- |
| 1 | Capillary refill rate | 0.0 | Categorical |
| 2 | Diastolic blood pressure | 59.0 | Continuous |
| 3 | Fraction inspired oxygen | 0.21 | Continuous |
| 4 | Glascow coma scale eye opening | 4 Spontaneously | Categorical |
| 5 | Glascow coma scale motor response | 6 Obeys commands | Categorical |
| 6 | Glascow coma scale total | 15 | Categorical |
| 7 | Glascow coma scale verbal response | 5 Oriented | Categorical |
| 8 | Glucose | 128.0 | Continuous |
| 9 | Heart rate | 86 | Continuous |
| 10 | Height | 170.0 | Continuous |
| 11 | Mean blood pressure | 59.0 | Continuous |
| 12 | Oxygen saturation | 98.0 | Continuous |
| 13 | Respiratory rate | 19 | Continuous |
| 14 | Systolic blood pressure | 118.0 | Continuous |
| 15 | Temperature | 36.6 | Continuous |
| 16 | Weight | 81.0 | Continuous |
| 17 | pH | 7.4 | Continuous |

Table 2. List of 17 clinical variables and their default imputation values for the MIMIC-III mortality prediction dataset.
