# Supplementary material for: Exploiting Missing Value Patterns for a Backdoor Attack on Machine Learning Models of Electronic Health Records: Development and Validation Study
Source: JMIR Med Inform. 2022 Aug 19;10(8):e38440. doi: 10.2196/38440 (PMC9440413; doi:10.2196/38440)
Supplement: Multimedia Appendix 2 [file medinform_v10i8e38440_app2.docx]

# Multimedia Appendix 2 of “Backdoor Attack on Machine Learning Models of Electronic Health Records: Exploiting Missing Value Patterns”

## Multimedia Appendix 2

### Detail Experiment Settings

In the “Discrimination Performance in Target Poisoning” section, we describe the targeted attack. To clarify the composition of the test dataset regarding the target, we show the number of target data that weighs more than 80kg in Table 3.

|  | **Positive group** | **Negative group** | **Total** |
| --- | --- | --- | --- |
| **# data** | 374 | 2,862 | 3,236 |
| **# Target data**  **(>80kg)** | 278 | 2,145 | 2,423 |
| **# Non target data**  **(<=80kg)** | 96 | 717 | 813 |

Table 3. The number of target data in the test dataset.

Since the composition of test datasets depends on attack objectives (i.e., false alarm or missing detection) and the type of data under a test (i.e., target data or non-target data), the precision of the random classifier accordingly changes. We summarize the precision in Table 4. The precisions are computed based on Table 3.

|  | **False alarm** | **Missing detection** |
| --- | --- | --- |
| **Target data** | 374 / (2145 + 374)  = 0.1484 | 278 / (2,862 + 278) = 0.0885 |
| **Non-target data** | 374 / (717+ 374) = 0.3428 | 96 / (2,862 + 96)  = 0.0324 |

Table 4. The precisions for several targeted attack settings.
